# The Adaptation of Digital Health Solutions During the COVID-19 Pandemic in Hungary: A Scoping Review

**DOI:** 10.34172/ijhpm.7940

**Published:** 2024-07-13

**Authors:** Bence Döbrössy, Edmond Girasek, Zsuzsa Győrffy

**Affiliations:** Institute of Behavioural Sciences, Semmelweis University, Faculty of Medicine, Budapest, Hungary

**Keywords:** Digital Health, Hungary, HER, COVID-19, Teleconsultations, Health Policy

## Abstract

**Background:**

Before the pandemic, digital health was merely a potential alternative to established systems of healthcare provision in Hungary. The technology was available but there was no governmental strategy regarding digital health use and development. The legal framework for digital solutions in healthcare was fragmented. COVID-19 created conditions in which digital solutions became the most feasible form of healthcare provision. We present the legislative and policy-making activities of the Hungarian government during the pandemic aimed at enhancing the organised and systematic use of these technologies.

**Methods:**

The core method used in our research is a review of legislation using the principles and methods of a systematic literature review. We searched the National Legislation Database for digital health related legislation for the period January 31, 2020 – June 1, 2022. To provide the context for the analysis, other relevant documents were identified through desk research.

**Results:**

Although established in 2017, the electronic health records (EHRs) were only widely used after the onset of the pandemic. Hungary used temporary state of emergency regulations to facilitate an increase in the number of teleconsultations. Our search identified 7 pieces of legislation that enabled healthcare providers to put digital solutions to instant use. They defined the conditions healthcare providers must meet for teleconsultations, set what interventions may be done, what can be reimbursed and dealt with security issues. The National Health Informatics Strategy of July 2021 is complex but mainly deals with technical issues. The three basic principles of the strategy are people centeredness, digital transformation and integrated care.

**Conclusion:**

Hungarian digital health solutions and policies fare well in international comparison. This is due to the intensive legislative activity of the pandemic period. The National Health Informatics Strategy ensures that the digital health solutions implemented during the pandemic outlive COVID-19.

## Introduction

###  Aims

**Study Highlights**

- The article presents the system of digital health in Hungary, focusing on its development as a response to the challenges of the pandemic.
- Before the pandemic the technology was in place to make the digital switch, but the fragmented regulatory system and lack of a clear legislative framework made its adaptation difficult.
- During the pandemic, the advantages of digital health solutions became more apparent.
- State of emergency decrees were used to create the environment in which digital solutions could be used.
- The National Health Informatics Strategy adapted in July 2021 provides the framework for digital health in the post COVID-19 era.

Our aim is to present the system of digital health in Hungary in terms of available services, policies, and legislation. Prior to 2021 Hungary had no national digital health strategy. Digital healthcare was expected to operate in a fragmented and incomplete legal environment. Although the technology needed was present, the legislation on how it may be used and what could be financed was lacking. COVID-19 created conditions in which social contact had to be limited as much as possible. Education, work, commerce, religious services, schools as well as healthcare provision had to go online as much as possible. “Tele-solutions” like online teaching, home office in work and telehealth-teleconsultations were needed to deal with the situation. For this paper we carried out a systematic review of digital health related legislation during the pandemic in Hungary to see what governmental efforts had been made to facilitate digitalisation in healthcare. We also looked at governmental strategy documents to see what the intended future direction of these newly adapted solutions may be. Hungary is usually not included in international digital health comparative studies, so this is a largely unmapped area.

###  Basic Concepts 

 The following World Health Organization (WHO) definition of Digital Health is used in this paper “*the field of knowledge and practice associated with the development and use of digital technologies to improve health. Digital health expands the concept of eHealth to include digital consumers, with a wider range of smart-devices and connected equipment. It also encompasses other uses of digital technologies for health such as the Internet of things, artificial intelligence [AI], big data and robotics*.”^1^

 We regard digital health as an umbrella concept that includes *eHealth*, indicating information and communication technology use for health, and *mHealth*, meaning mobile technology use for health. *Telehealth*, ie, the remote provision of care and monitoring for patients with digital tools, is also integral to the concept. Big data created by these technologies is a further possibly important benefit and an essential component of digital health.^2^

###  Background

 Long before COVID-19, the systematic implementation of digital tools had been a priority for both the European Commission and the WHO.^3,4^

 Despite evidence of the benefits of digital solutions in healthcare in terms of quality, access and cost, only a number of countries made headway in it before the pandemic.^5^ Although by 2015 around 70% of European Union (EU) countries surveyed by the WHO had eHealth strategies, less than 30% had one related to telehealth. 69% of WHO Euro countries had legislation for electronic health records (EHRs), and even fewer on legal jurisdiction, liability, finance or big data use.^2^ As for implemented digital solutions, there were many pilot projects but less was offered on a nationwide, organised level.^6^ The new technology was available but the cultural and systems change that must accompany it was lacking.^7^ From a legislative and policy perspective, Hungary belonged to the lesser digitalised countries. Although legislation relating to EHRs was passed in 2016, the system was scarcely used. No laws, regulations and legislations were in place to govern teleconsultations. There was a system of ePrescriptions, but its use was cumbersome and in need of reform.

 The pandemic turned what had been perceived as a possible futuristic opportunity into a working solution to a current problem. Digital health used to be an unconventional alternative to traditional healthcare. It became more appealing in times when the directive was “stay at home.” The fragmented and incomplete legal environment had to be addressed very quickly so as to make legally applicable what was technically feasible.

 Two important features of digital health in the pandemic were telehealth and information provision. Patients had limited access to healthcare providers, so teleconsultations and ePrescriptions were used to bridge the physical gap. Lay people had inadequate information about COVID-19 so social media was utilised as an educational platform. Infodemics, the spread of false information, was a burning problem, so health authorities had to turn to online channels to combat it. Prescribing medication and the sharing of health-related documentation had to be digitalised so as to limit face-to-face contact. What must be realised however is that the above-mentioned issues were not new. They had existed even before the onset of the pandemic. Access to healthcare had always been a problem due to regional inequalities. The need for a channel of health education through which masses can be reached was a challenge even before COVID-19.^8^ So while the declared intention was to deal with the current problem posed by COVID-19, the solutions put in place had the potential to alleviate age old issues, too.

 In its third global survey on eHealth, the WHO lists the existence of relevant programmes, policies and regulation, funding, EHR and training as necessary prerequisites for the implementation of digital health.^9^ The truth of this was driven home during the pandemic when policy had to be made in forced march mode.

 Regulation must deal with issues ranging from cyber security and privacy, clinical quality control, acceptable technical standards and financing, and reimbursement. Funding is necessary for digital health tools, as well as for facilitating the organisational and systems changes. Policies must regulate reimbursement for digital services as it will motivate healthcare providers to engage in them. Users must be empowered to make the best of digital services. Training of professionals and user-friendly interfaces are key aspects here.^10^

 Our aim was to look at how these conditions were ensured by COVID-19 era decrees and regulations in Hungary.

## Methods

 What we are about to present in this paper is a narrative analysis with the approach of a systematic literature review. A similar approach was taken by Roziqin et al in their analysis of Indonesian government policies against COVID-19.^11^

 In our research we aimed to find all relevant legislation passed in Hungary during the COVID-19 pandemic pertaining to digital health. Hence, this is not a systematic literature review, but a systematic review of legislation documents using the principles and methods of a literature review. We reviewed governmental legal documents, not scholarly articles and studies. The documents our search targeted were new laws and the modification of previously existing laws, governmental and ministerial decrees, normative decisions and normative instructions.

 The database searched was https://njt.hu/. This is the *National Legislation Database* (Nemzeti Jogszabálytár) where legal and official announcements, laws, and regulations are published. Every piece of legislation is available on this site. It can be searched by key terms. The document type and the period during which it came into effect can also be set in the search engine.

 Search terms were in Hungarian as that is the language the documents were written in. The search terms we used were “digital health” (digitális egészség) “telemedicine” (telemedicina) “Teleconsultations” (távorvoslás) and “electronic health records” (EESZT).

 The starting date of the search period was January 31, 2020. This is when the Operative Board responsible for coordinating COVID-19 related activities was set up by the Hungarian government. This may be considered to be the first step in governmental efforts to prepare for the pandemic. For the end date, we chose June 1, 2022 when the COVID-19 related state of emergency governance was terminated.

 The database was searched by BD, ZG, and EG. All documents were read in full by all three authors. The authors together established the inclusion criteria discussed below and the framework of interpreting the documents. Decisions about inclusion were made jointly. The documents were scrutinised for their relevance to the use of digital health solutions.

 Altogether our search came up with 60 laws (including modifications of previously existing laws), governmental and ministerial decrees, normative decisions, and normative instructions. After removing duplicates 47 documents remained. Duplicates happened because the same document was found through more than one search term. Sometimes the same issue was addressed on different levels of the legislative hierarchy. For example, the issue of Videotechnology Facilitated Teleconsultations with Possible Face Recognition was addressed as a law and also by governmental and ministerial decrees.

*Inclusion criteria *– the legislation had to pertain to digital health, telemedicine, or the functioning of EHRs. If it just applied previous legislation to special populations (for example professional soldiers, refugees, people without health insurance number) it was not included in the analysis. We also excluded modifications of laws where our keywords were mentioned, but the modifications did not relate digital health.

 Finally, as the paper is about the acceleration of the use of digital solutions in healthcare during COVID-19, and not COVID-19 per se, legislation that had no relevance beyond COVID-19 was also excluded.

 After excluding the legislations which did not meet the criteria, 7 documents were left.^12-18^ These form the core of our analysis.

 Besides the legislations, other types of material were also used in preparing this paper.

 Three important current governmental strategy documents were identified concerning the future of digital health in Hungary. These are (a) For a Healthy Hungary 2021-2027 – Healthcare Sectoral Strategy – Ministry of Human Resources, January 2021,^19^ (b) National Health Informatics Strategy accepted in July 2021,^20^ and (c) National Digitalisation Strategy (NDS) 2022-2030.^21^ They are discussed in more detail as they contain governmental and ministerial strategy concerning digital health development.

 Data on the digital readiness of Hungary comes from the Digital Economy and Society Index (DESI). The DESI summarises indicators on the digital performance of EU countries. It measures the overall digital performance of EU member states. As it is done annually, performance and progress may be tracked. Analysis is done on 3 levels. On the first level the index consists of five main indicators: Connectivity, human capital, use of internet, integration of digital technology, and public digital performances. At the second level these indicators are divided into 13 subgroups which are further divided into 34 subgroups of the third level.

 Data on health status and telemedicine use comes from the Organisation for Economic Co-operation and Development (OECD) 2021 Country Health Profile. This is a reliable data source frequently used by studies to make cross country and longitudinal comparison. Data on EHR use comes from governmental sources.

**Figure 1:**
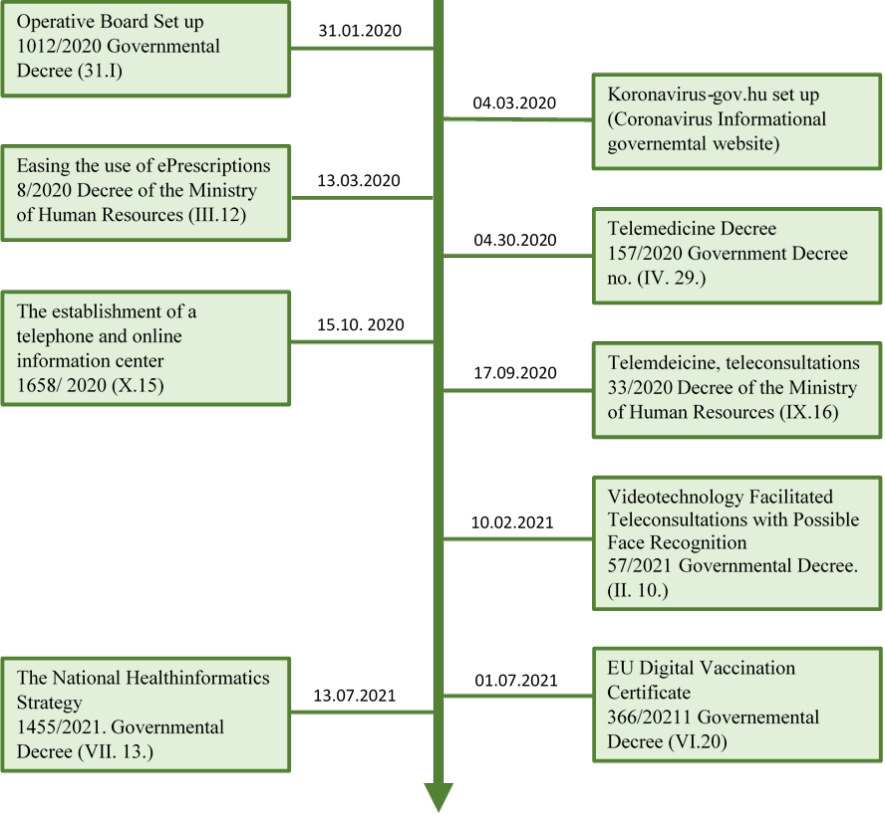
Digital Health Related Decrees and Legislations Passed During the Pandemic.

Figure 1 depicts the timeline of the major legislations related to digital health during the pandemic.

## Results

###  Digital Health Readiness in Hungary – the Present

 In discussing our results, we proceed with a short discussion of the health status, healthcare system and digital readiness of Hungary as this context is important for the understanding of the possibility of digital health implementation.

####  Country Context: Healthcare and Health Status

 Unless otherwise referenced, all data in this section is from the OECD 2021 Country Health Profile, Hungary.^22^

 Life expectancy is 75.7 years which is low for a country with Hungary’s economic development. It is almost 5 years lower than the EU average. Health spending as a proportion of gross domestic product is also low, at 6.4% compared to the EU average of 9.9%. Hungarian per capita health expenditure is less than 50% the EU average after adjusting for differences in purchasing power. Amenable mortality is higher than the European average.

####  Digital Readiness

 Based on their current digital habits, the population of Hungary is ready to upscale their use of digital health technologies. As of January, 2021, 83% of the population were internet users and 73.5% were active social media users.^23^

 The Eurostat regional Yearbook 2021 reports a significant regional divide in internet use in Hungary. While daily internet use among 16-74 year olds is 89% in Budapest, it is only 72% in the northern region of Hungary.^24^

 Internet availability is only one precondition of digital health application. People must have the skills and confidence to operate the system, ie, they need digital health literacy.

 There is evidence that the internet is an important source of health-related information for many in Hungary. Indeed, according Girasek and colleagues’ research done on a representative, large sample, 81.3% of the respondents reported using the Internet in general, with almost 90% of them also using it for health and illness related issues. This means that the frequency of health-related internet searches is very high. Nearly half of those surveyed would try a teleconsultation and would like to be recommended reliable websites, apps and sensors by their doctor.^25^

 According to the 2021 DESI, Hungary performs well in 5G readiness, overall fixed broadband take-up and 100 Mbps take-up.^26^ 89% of households had fast broadband in Hungary. Connection speeds were very good, 56% of homes had at least 100 Mbps fixed broadband compared to the EU average of 34%. Hungary scored low on Human capital. Only 49% of the population had at least basic level digital skills compared to the EU average of 56%.

####  Telehealth 

 As early as 12 March, even before lockdown was initiated, the *8/2020 Decree of the Ministry of Human Resources (III. 12) on easing the use of ePrescriptions* came into being, in order to make the use of ePrescriptions easier.^17^ Although ePrescriptions were available before, their use was cumbersome as it was very difficult for the person in whose name the prescription was made out to delegate authority for others to pick up the prescription from the pharmacy. In the case of sick, elderly people, this is a real issue.

 Telemedicine use in Hungary is governed by law created for the state of emergency during the COVID-19 epidemic. State of emergency rules governing teleconsultations came into force on April 30, 2020 when the government issued *Government Decree no. 157/2020 (IV. 29.)* known as the *Telemedicine Decree*.^12^ Healthcare facilities were obliged to be equipped to provide teleconsultations. They had to have a protocol and patient information material for it. The decree defined the criteria of teleconsultations as well as the aims it must serve. It permitted using telemedicine to set up diagnoses, suggest therapy, consult, manage and direct patients, give referrals, and prescribe medication. The following telemedicine interventions were reimbursed by the National Health Fund: control examination, consultation, electroencephalogram with telemetric, electrocardiogram (ECG) with telemetric, transtelephonic ECG use in acute cardiac cases, transtelephonic ECG in postoperative cardiac cases, transtelephonic ECG in elective cases, transtelephonic ECG in postoperative cardiac cases during emergency interventions, the preparation, and sending of samples sent by telepathology in colorectal screening, the evaluation of samples sent by telepathology in colorectal screening, supplementary points for second opinion during colorectal screening, dental teleradiology, pain monitoring and its computerised evaluation, documented psychiatric consultation on the telephone.

 Very much related to this is the *33/2020 Decree of the Ministry of Human Resources (IX.16) on Telemedicine and Teleconsultations*.^14^ The decree stated that the healthcare provider must possess the following to be allowed to provide telemedicine services: information technology (IT) equipment needed for the service provision, medical equipment needed for the provision, detailed telemedicine use guidelines and patient information sheets, broadband, stable internet and virus protection. The decree also established what telemedicine interventions could be reimbursed.

 A related decree passed in the period is *governmental decree 57/2021. (II. 10.) on Videotechnology Facilitated Teleconsultations with Possible Face Recognition*.^16^ This permitted doctors to diagnose, prescribe, and propose therapies as a result of teleconsultations using video-technology where facial identification is feasible. Facial recognition is needed only if no other method is possible due to the nature of diagnoses or data protection. Sufficient identification documents must be shown during the consultation.

 Another piece of legislation vital for digital health is the establishment of the 7/24 “health-line” (Egészségvonal). This is a free online and telephone hotline health information centre which came into effect on October 15, 2020. *This is 1658/2020. (X. 15.) Governmental Decree on The National Healthcare Telephone Customer Service and Online Information Center*.^18^ The centre is operated by the National Public Health Centre (Nemzeti Népegészségügyi Központ). This initiative goes beyond COVID-19. It contains all sorts of health and illness related information.

 The free of charge telephone lines are answered 7 days a week, 24 hours a day by trained non-medical dispatchers. They can inform callers on COVID-19 related issues, health services and facilities, the use and functions of the EHRs, ePrescriptions, screening services, prevention and health promotion.

 The webpage contains information on healthy lifestyle, screening tests, vaccinations and health promotion. It lists health facilities by type and location. There is also an A-Z of symptoms and illnesses written in a clear, easy to understand form.

 Although the service is not widely known it has the potential to be very useful by providing easy to understand, reliable health related information. It is easy to reach even for the less digitally literate through the toll-free telephone numbers. It has the potential to relieve pressure on health services.

####  Electronic Health Records: Features of the Hungarian National eHealth Infrastructure

**Figure 2:**
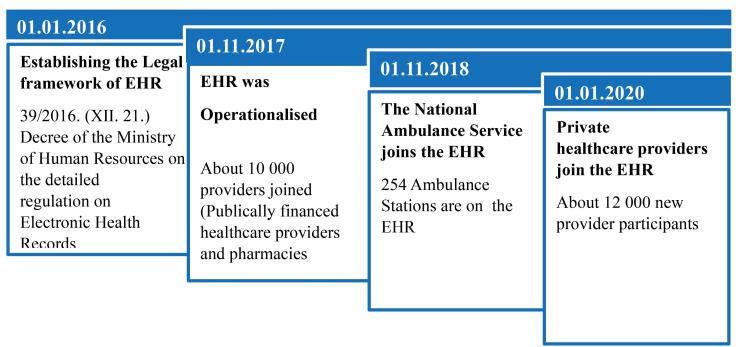
Timeline of the Development of Electronic Health Records in Hungary. Based on Varga et al.^27^ Abbreviation: EHR,electronic health record.

Figure 2 depicts the timeline of the most important elements in the development of the EHRs in Hungary.

 The functioning of the EHR was codified in the *39/2016. (XII. 21.) Ministry of Human Resources decree on the detailed regulation of the National eHealth Infrastructure.*^28^

 The EHR, a cloud-based communication interface system, provides a communication space for providers and patients. Healthcare facilities are obliged to be connected to it. During the pandemic, more than 20 new functionalities were implemented, including collecting and reporting pandemic-related data, ordering protective materials and devices, vaccine passports, booking vaccination appointments, teleconsultations support and a Home Quarantine Support App.^27^ Before registering, private and public healthcare providers and pharmacies must undergo an eLearning training course and pass an exam. An estimated 75 million medical documents and close to 180 million doctor-patient appointments are recorded online in the system annually.^27^ The figure went up from 8000 prescriptions a day before the pandemic to 800 thousand by 2021. The data indicates that in 2018, medical professionals performed approximately 8.6 million procedures utilizing the system. This number increased to 17.6 million procedures in 2019, and reached 30 million procedures in 2020.^27^

 Data must be recorded on three separate modules: Central Event Catalogue (outpatient and inpatient specialised care, general practitioner and paediatric care, ambulance car care, emergency room event care), Medical Documents (discharge summaries, outpatient medical reports, general laboratory results, microbiology laboratory results (optional), computed tomography and magnetic resonance imaging reports, other imaging reports, surgical procedure reports, ambulance car reports and specialist referrals) and medical profile (data on the general health condition of the patient).

 The following are the central EHR functions:

- *eProfile*: A record of the general health status and health characteristics of the individual that may affect future treatment.
- *ePrescription*: Besides facilitating a contact-free prescription, this module has a lot of vital features for both doctor and pharmacist. Physicians are allowed to check the prescription history of patients they are treating. Certified pharmacists are also able to check dispensing history and check for possible interactions (depending on consent rules).
- *eReferral: *This module creates the communication between the IT system of the referring physician and the physician performing the examination, guaranteeing the reliable transfer of all relevant information.

###  Digital Health Strategy in Hungary – the Future

 In response to its relatively slow rate of overall digital development, Hungary adopted the NDS 2022-2030, in the fall of 2021.^21^ The pillars of the strategy are digital infrastructure, digital skills, digital economy, and digital state, corresponding to the DESI dimensions. Digitalisation of public administration is a relevant feature of the NDS prioritising (1) synchronised, user-centred digital development of administrative and professional systems; (2) launching a data-driven administration; (3) developing smart settlements and smart areas; (4) improving security of government electronic services; and (5) digitalising public services in healthcare, transport, energy, education, and culture. Measures were introduced to improve population digital competence including IT curriculum reform, adult training programmes with a special training programme for teachers, and the distribution of IT equipment to those in need of it. The NDS set up large-scale programmes for citizens highlighting interventions for social inclusion and e-health (improving the competence of both citizens and healthcare workers).

 Another significant strategy document for the future of digital health in Hungary is the *For a Healthy Hungary 2021-2027 – Healthcare Sectoral Strategy – (Ministry of Human Resources, 2021, January)*.^19^

 This is the first ever national health strategy in Hungary that features digital health. The following are the basic pillars of the eHealth action plan: people centred eHealth, regulated processes, data-based decisions, unification of system level IT, digitalisation of the process of care provision, ePublic administration, support of eGovernance, and creation of the institutional system of eHealth.

 This general health sector strategy laid the groundwork for *The National Health Informatics Strategy* accepted by Governmental Decree 1455/2021. (VII. 13.). This document is discussed in more detail here.^29^ The government has accepted Governmental Decree 1455/2021. (VII. 13.) on the National Health Informatics Strategy to improve healthcare and public health by using the tools offered by informatics, digitalisation and AI. The three basic principles of the strategy are people centeredness, digital transformation and integrated care. The focus is broad, encompassing not only healthcare system management, but also disease prevention and health promotion. The specific areas the strategy targets are discussed below. It is important to stress that what we are about to discuss is not the present state of digital health but the strategy for the near future.

####  The Development of eHealth Awareness (eHealth Functions for the Population, Education, and Prevention)

 Good, easy to understand information is essential for disease prevention and health promotion. The aim is to provide effective and credible information sources such as a call centres and chat-bots integrated into the EHR.

 People will be encouraged to develop their eProfile on EESZT by keeping online health logs, in which they record health and illness related data. The systematic use and analysis of the mHealth data individuals upload will also be an important public health resource. A centralised EHR-based mHealth app is in the pipeline. Lay and professional digital health training courses will be offered to increase digital literacy. Based on their eProfile, patients can receive personalised information on illness management programmes.

####  Digitalising Processes of Care

 Telemedicine and teleconsultations could help improve access to healthcare in more remote areas. The strategy aims to use this technology to improve the organisation of patient pathways. It is hoped that managing documentations digitally, mostly through EHR, will reduce the administrative tasks of doctors. The development of health informatics infrastructure and data storage and management capacities is key to this. Centralised patient management services, the centralised management of waiting lists and in and out-patient qualification systems will also be managed digitally.

####  Health System Managements

 The strategy plans to include accounting, analytical operations and controlling in the integrated digital system. Its aim is to help both the central and the service provider level in making evidence based, cost-effective decisions while reducing the pressure on human labour. The digital system will be an effective tool in linking data management and analysis on the provider side with third-party financers and researchers. Data protection and issues of privacy must be maintained in analytical data-linking. A central accreditation agency is to be set up to formulate accreditations rules and requirements.

####  Telemedicine

 The strategy envisions a centralised system integrated into the EHR which is capable of video and voice communication as well as handling the relevant administration. Virtual appointments could be booked here, too.

 The aim is to establish a nation-wide telediagnostic centre with state of the art analytical and diagnostic services. AI-based telediagnostic services are planned.

 The strategy promotes centralised telecare and telemedicine development. The centre will be able to provide 24-hour services available from anywhere in the country.

 Telecare services will also be offered for the management of chronic conditions. The strategy aims to limit the number of face-to-face doctor-patient encounters.

####  Big Data

 Big data generated by the EHR can be used for diagnostic, screening, macro level public health and scientific research benefit as well. Big data use depends on adequate anonymising and controlling. Professional registries will be merged and there will be established processes of EHR data utilisation for research.

Table provides a summary of the services proposed by the Health Informatics Strategy.

**Table:** Services Proposed by the Strategy – Summary

**General Service Type**	**Components of the Service**
Population services	Information Centre, National Healthcare Call Centre, chat-bot- automatised diagnostic system, centre app and web-surface, mobile app integrated to the call centre, catalogue of services
Support services for healthcare providers	Healthcare portal and app, speech recognition-based documentation
E-administration	Fee for services management, management of medical certifications needed for public administration
Knowledge base database	Quality controlled information source for lay and professional people
Telemedicine	Virtual medical office, teleconsultations, telediagnosis, telecare, and telemedicine on duty
Prevention	New public health register, continuous recording and registering of patient health data, digital support of healthy lifestyle, screening support, and managing vaccinations
Support for central health administration	National laboratory database and system, pathology information system, monitoring system for one day surgery, accreditation system for in and outpatient care
Public health developments	Epidemics system, public health laboratories, medical induced radiation management, work permit system
Managing developments in medical specialities	Developing registries, professional supervisory bodies, digital imaging anonym databases, tumor bank, diagnostic imaging databank for telemedicine for cancer patients, clinical databases, virtual onco teams
Development of specialist system financing	National Health Insurance Fund Management, cybersecurity
Research and development	eHealth technology transfer, pilot projects, clinical research support system
AI	Integrating AI diagnostics into professional systems, AI in screening programmes
Financial and controlling tasks	Data analytical system, human resources managing system, central economic management monitoring
Methodology	Central and professional methodology
Training	EHR related training, eHealth training, training and accreditation system
Central database	Development of the data collecting branch of the data analytical system, system of primary and secondary use of databanks

Abbreviations: EHR, electronic health record; AI, artificial intelligence.

 The operationalisation of the strategy centres on the *Support for The Digital Reorganisation of Healthcare Recovery and Resilience Facility 8.3.1-22* project.^30^

 Within its framework, programmes can apply for over 132 billion HUF non-refundable support.

 It is specified that participants may only include the National Public Health Centre, The National Institute of Pharmacy and Nutrition, National Healthcare Service Centre, The National Ambulance Service, and the National Institute of Oncology.

 Applications are sought in the following areas: (*a*) development of telemedicine through the establishment of a teleconsultation and telecare centre, (*b*) development of eHealth conscious society through training, sensitising and the establishment of population eHealth functions, (*c*) further development of EHR and related systems, (*d*) reducing the administrative burdens of doctors, (*e*) developing and putting health data to use, unified data analysis EHR research centre, (*f*) developing the security of the healthcare provision infrastructure, professional data collection and development of central registries and professional processes and the development of central institutions.

## Discussion – Where Does Hungary Stand in International Comparison?

 In 2019 the Bertelsmann Stiftung-Empirica research institute compared and ranked the digital health maturity of 17 countries using a Digital Health Index it created and concluded that success in digitalising healthcare is built on effective strategy, strong political will, a well-articulated national mandate and dedicated agencies.^31^ The WHO also stresses that relevant programmes, policies and regulations are the prerequisites for the implementation of digital health.^32^

 Before COVID-19 none of these requirements were met in Hungary. Digital health was not mentioned in the strategies of the health sector and relevant laws and legislations were scarce and fragmented. Even those doctors who might have been keen on using digital health solutions were discouraged by the absence of supportive conditions.

 The raison d’être of the governmental and ministerial decrees on digital health passed in quick succession was not the realisation that digitalisation is needed to improve quality of care, accessibility, safety, and equitability. Instead, it was to facilitate the provision of contactless healthcare as much as possible. The *8/2020 Decree of the Ministry of Human Resources (III.12) *eased the use of ePrescritions*. Government Decree no. 157/2020 (IV. 29.) *known as the Telemedicine Decree, regulated the conditions under which healthcare facilities may provide teleconsultations. It obliges facilities to have a teleconsultation protocol and discusses what interventions may be provided online. *The 33/2020 Decree of the Ministry of Human Resources (IX.16) on Telemedicine and Teleconsultations* discusses the type of equipment needed for the services. *Governmental decree 57/2021. (II. 10.) on Videotechnology Facilitated Teleconsultations with Possible Face Recognition *dealt with immediate security issues. The* 1658/2020. (X. 15.) Governmental Decree on The National Healthcare Telephone Customer Service and Online Information Centre *led to the establishment of a 24/7 toll-free telephone centre and easy to read, quality control webpage. This is important to combat infodemics while easing pressure on healthcare providers. Through these legislations, policy makers defined financial mechanisms, adjusted reimbursement for telecommunication to make it more attractive for doctors, created guidelines and removed restrictions. This is not unique to Hungary, it was practiced in numerous European countries.^22^

 Today the conditions are there to facilitate digitalisation in healthcare. The EHR and ePrescriptions are widely used and understood. As stated earlier, the number of procedures initiated on EHR quadrupled from 2018 to the time of writing COVID-19 gave both doctors and patients experience and familiarity with digital health. The Health Informatics Strategy and the Health Sectorial Strategy have clearly placed digital health on the agenda.

 Nevertheless, there are still areas where digital health must be improved. First, big data utilisation is not advanced. Second, telehealth is not integrated in the EHR system, but it will be by 2027. Third, the EHR does not have appointment booking and health education functions, but they are to be implemented by 2027 according to the national strategy. The booking function is ready but it has only been activated for COVID-19 vaccinations. The system of ePrescriptions is fully functional, widely used and socially accepted.

 The privacy of patients is safeguarded in the system with adequate patient and doctor personal identifiers. With the exception of emergencies, patients can determine who has access to their data.

## Conclusion

 There is international evidence that digital healthcare solutions lead to more equitable and efficient healthcare.^6^ Hence the rapid legislative and strategy making process discussed may have wide ranging significance beyond COVID-19. The digitalisation, health informatics and health sector strategies discussed in our paper outline action plans that will hopefully help alleviate age old issues like regional inequalities and difficulties in accessing healthcare.

 The deployment of rules and regulations to facilitate telemedicine and eHealth during the pandemic also served to speed up doctors’ and patients’ cultural acceptance of these solutions. COVID-19 has led to increased digital use in the areas of communication through apps, websites and sharing health-related information on social media. The major examples here are contact tracking, monitoring and surveillance, supporting symptom tracking and self-diagnosis, and the use of apps to support self-isolation.^2^ Vaccination-related digital solutions proved to be another relevant area. Managing vaccine appointments, identifying individuals eligible for vaccination, motivating people to take vaccinations, issuing vaccination certificates to help reopen the economy are some of the most important new digital solutions.^33^

 During COVID-19, health professionals and patients were eager to use digital technologies as it was a safe alternative to physical contact. However, with the return to normalcy, system-level backing of the continued use of digital health tools on a daily basis is important as face-to-face interactions are available again. Adequate reimbursement, training and clear guidelines will ensure continued provider motivation so that digital technology will not be seen as a “poor substitute of the real thing.”

 EU level coordination and exchange of experience is important. Here again, in developing the Hungarian strategy, international experience was utilised.

 The question arises whether these legislations would have been passed anyway without COVID-19. In answering this hypothetical inquiry we must remember that the rapid acceleration in digital health use and legislation during COVID-19 was not unique to Hungary.

 Before COVID-19 there was great discrepancy in digital health maturity among various countries, but during COVID-19 increase in use was more or less universal. So, although it is not conclusive, we may assume that the sudden increase in digital health-related legislative and regulatory activity was due to the conditions created by COVID-19 responses, mainly social distancing whose impact on healthcare provision was partly overcome by telemedicine.

 The paper suffers from a number of limitations. Sources were scarce. Some strategies the decrees were based on are unpublished, there is a lack of professional and academic analysis of them and there is no evaluation of COVID-19 era digitalisation in healthcare.

 The case of Hungary provides evidence that the new technology must go hand in hand with a shift in the culture of healthcare provision and reception. Digital health will only fulfil its promise, if doctors and patients are willing to use it. COVID-19 “forced” the providers and recipients of healthcare to utilise digital solutions. Policy-makers were quick to act in creating the financial and legal conditions to facilitate this during the emergency environment. The challenge now is to create the conditions conductive to the institutionalisation of digital health in the post COVID-19 era, too.

## Ethical issues

 Not applicable.

## Competing interests

 Authors declare that they have no competing interests.
